# Task-Dependent Differences in Operant Behaviors of Rats With Acute Exposure to High Ambient Temperature: A Potential Role of Hippocampal Dopamine Reuptake Transporters

**DOI:** 10.3389/fnbeh.2019.00015

**Published:** 2019-02-04

**Authors:** Shuo-Fu Chen, Chuen-Yu Chuang, Chih-Chang Chao, Yi-Hua Yang, Chi-Yun Chu, Chang-Yu Yao, Yu-Chen Su, Ya-Huei Huang, Ruey-Ming Liao

**Affiliations:** ^1^Institute of Neuroscience, National Cheng-Chi University, Taipei, Taiwan; ^2^Department of Psychology, National Cheng-Chi University, Taipei, Taiwan; ^3^Affiliated High School, National Cheng-Chi University, Taipei, Taiwan; ^4^Research Center for Mind, Brain and Learning, National Cheng-Chi University, Taipei, Taiwan

**Keywords:** warm ambient temperature, schedule-controlled behavior, FR-typed, DRL-typed, brain dopamine

## Abstract

Behavioral or cognitive functions are known to be influenced by thermal stress from the change in ambient temperature (Ta). However, little is known about how increased Ta (i.e., when the weather becomes warm or hot) may affect operant conditioned behavior and the neural substrates involved. The present study thus investigated the effects of high Ta on operant behaviors maintained on a fixed-ratio 1 (FR1) and a differential reinforcement for low-rate responding 10 s (DRL 10-s) schedule of reinforcement. The rats were randomly assigned to three groups receiving acute exposure to Ta of 23°C, 28°C, and 35°C, respectively, for evaluating the effects of high Ta exposure on four behavioral tests. Behavioral responses in an elevated T-maze and locomotor activity were not affected by Ta treatment. Regarding operant tests, while the total responses of FR1 behavior were decreased only under 35°C when compared with the control group of 23°C, those of DRL 10-s behavior were significantly reduced in both groups of 28°C and 35°C. Distinct patterns of inter-response time (IRT) distribution of DRL behavior appeared among the three groups; between-group differences of behavioral changes produced by high Ta exposure were confirmed by quantitative analyses of IRT data. Western blot assays of dopamine (DA) D1 and D2 receptor, DA transporter (DAT) and brain-derived neurotrophic factor (BDNF) were conducted for the sample tissues collected in six brain areas from all the subjects after acute high Ta exposure. Significant Ta-related effects were only revealed in the dorsal hippocampus (dHIP). In which, the DAT levels were increased in a Ta-dependent fashion that was associated with operant behavior changes under high Ta exposure. And, there as an increased level of D1 receptors in the 28°C group. In summary, these data indicate that the performance of operant behavior affected by the present high Ta exposure is task-dependent, and these changes of operant behaviors cannot be attributed to gross motor function or anxiety being affected. The regulation of dHIP DAT may be involved in this operant behavioral change under high Ta exposure.

## Introduction

In addition to physiological responses monitored by the central thermoregulation system (Nakamura, 2011), behavioral function is influenced by thermal stress resulting from the changes in ambient temperature (Ta; Cheshire, 2016). Notably, behavioral performance affected by Ta can be more diverse and unpredictable than the thermoregulation processes rigidly controlled by certain levels of brain mechanisms (e.g., hypothalamic or medullary). While previous studies showing the effectiveness of varied Ta’s at behavioral level have mostly recruited the test models based on reflexive system (Bouali et al., 1995; Gallup, 2010; Suwanapaporn et al., 2017), studies on the effects of Ta on the associative conditioning behavior paradigms are scarce. It is, thus, important to evaluate whether the conditioned behavior can be influenced under a non-thermoneutral environment. Individuals and homoeothermic animals living in the tropical regions frequently face high Ta when exposed to excessive natural heat and heat stroke. To date, how different degrees of high Ta may affect the operant conditioned or schedule-controlled behavior and its underlying neural mechanisms is still largely unknown (but see Barofsky, 1969; Thomas et al., 1991).

In consideration of the functional relationship potentially existing between the operant behavior and high Ta, the present study was designed to assess the effects of high Ta exposure on the performances of operant behaviors maintained in a fixed-ratio 1 (FR1) and a differential reinforcement for low-rate responding 10 s (DRL 10-s) schedule of reinforcement in the rat. Operant behaviors trained on these two different schedules of reinforcement are distinctively characterized by not only the task difficulty but also the behavioral component or psychological construct (Ferster and Skinner, 1957). That is to say, behavioral inhibition and timing process are especially required for the subject to perform on the DRL-typed behavior (Kramer and Rilling, 1970; Sanger and Blackman, 1975; Neill, 1978; McClure and McMillan, 1997; Bayley et al., 1998; Monterosso and Ainslie, 1999; Paule et al., 1999; Cheng et al., 2006). Thus, unlike a relatively high response rate that is generally measurable in FR-typed schedule, a low-rate responding on operant manipulandum is typically elicited by DRL-typed schedule. A previous study has shown that dissociable effects appear in operant behaviors maintained on DRL and FR in the rat under stress of tail-pinch and psychoactive drug treatments (Chang et al., 2000). Based on the premise that temperature stress causes a physiological response with a rapid rise in body temperature in rats exposed to warm or cold environment (Long et al., 1990), the effects of high Ta exposure on these two operant behaviors are expected to be distinctively different. With regard to the range of warm/hot Ta being examined, the relatively high degrees of warm Ta exposure, e.g., 36–40° Celsius (°C), have been shown to greatly affect behavioral manifestation (Carlisle and Laudenslages, 1976; Bouali et al., 1995). Furthermore, behavioral and autonomic thermoregulations have been shown to be different in mice following exposures to mild and severe heat shock (Leon et al., 2010). Accordingly, 28 and 35°C were chosen in this study as two different degrees of high Ta to be compared with the control Ta of 23°C. The acute Ta exposure was manipulated to determine the effects of different levels of high Ta on DRL- and FR-typed operant behaviors. In addition, the anxiety-like response in an elevated T-maze and the locomotor activity after the acute exposure to high Ta were assessed. The elevated T-maze has been used to examine anxiety-like behavior in rodents and is validated by pharmacological tests showing the avoidance attenuated by anxiolytic drugs (Graeff et al., 1998; Zangrossi and Graeff, 2014). Whether acute exposure to high Ta would alter anxiety-like response on the elevated T-maze is currently poorly understood.

Substantial evidence has been accumulated indicating that a functional relationship exists between stress and brain dopamine (DA) systems (Roth et al., 1988; Feenstra, 2000). Considering that exposure to high Ta is a kind of stress, physiological and neurochemical functions of DA can correspondingly respond as a part of thermoregulation for the subject during environmental temperature challenge. Indeed, several studies have shown that experimental manipulations of DA receptors and the release and reuptake of DA can produce thermoregulatory responses in mammals (Cox and Lee, 1977, 1980; Cox et al., 1978; Brown et al., 1982; Lin et al., 1982, 1992, 1995; Lin and Tsay, 1985; Chaperon et al., 2003). Neurotrophins, including brain-derived neurotrophic factor (BDNF), are altered in stressful conditions involved with psychosocial and physical factors (Alleva and Santucci, 2001; Alleva and Francia, 2009). Assuming that high Ta exposure may act as a stressor and affect the DA- and BDNF-associated brain regions, we collected tissues from six brain areas that were subjected to Western blot assay to determine the protein expression of DA D1 and D2 receptors (D1R and D2R, respectively), DA transporter (DAT), and BDNF. The brain areas included the medial prefrontal cortex (mPFC), dorsal striatum (dSTR), nucleus accumbens (NAcc), amygdala (AMG), dorsal hippocampus (dHIP) and hypothalamus (HYPO).

The objective of this study was to examine the effects of high Ta on a FR and a DRL operant tasks along with anxiety-like response in an elevated T-maze and the locomotor activity. In addition, Western blot assays of D1R, D2R, DAT, and BDNF were conducted for the sample tissues collected in six brain areas from all the subjects following acute high Ta exposure after the end of behavioral tests.

## Materials and Methods

### Subjects

Eighteen male Wister rats (BioLASCO Taiwan Co., Ltd.), averaging approximately 250 g of body weight and 6 weeks old upon arrival, were housed individually. The rats were handled daily and allowed 10 days of acclimation to the colony. Food and water were provided *ad libitum*, except for the experiments of operant behavior. The rats were maintained on a water restriction regimen such that there was 5 min access to tap water in the home cage occurring no sooner than 30 min after the end of each daily experimental session of operant behavior. During this period, the body weight was monitored and allowed to gain weight throughout the course of operant experiment on a delayed-growth curve. Food pellets were continuously available in each home cage. Training and/or test sessions were conducted daily at the same time (10:00–15:00) during the light portion of the vivarium’s 12 h/12 h light/dark cycle (lights on at 7:30). The temperature of the colony and the behavioral test room was maintained at 23 ± 1°C throughout the experiment. All procedures were conducted in accordance with the NIH Guide for the Care and Use of Laboratory Animals and approved by an institutional review committee of animal use and care at National Cheng-Chi university.

### Apparatus

Operant behaviors were measured using a custom-made operant system with four chambers located in a room separate from the animal colony. The interior dimensions of each chamber were 20 cm × 25 cm × 30 cm (MED Associates Inc., St. Albans, VT, USA). Aluminum panels formed the front and back walls, and clear Plexiglas comprised the remaining sides and the top. Stainless steel rods (with a diameter of 5 mm) were set 11 mm apart to provide flooring. Each chamber was equipped with a lever positioned 7.3 cm above the floor and 4 cm from the right corner of the front panel. A liquid dispenser was set outside of the front panel of the chamber. The reinforcer delivery mechanism provided 0.04 ml of tap water at each presentation. The water was delivered into a receiving dish (25 mm diameter) located at the center of the front panel and 2 cm above the floor. The chamber was illuminated by a small light bulb located 10 cm above the floor and positioned 5 cm from the left corner of the front panel. Each chamber was enclosed in a plywood box with a fan to provide the necessary ventilation and to mask any outside noise. The four operant chambers were serviced and controlled by a microcomputer with an in-house designed program to control the operant environment as well as to allow data collection (Cheng and Liao, 2007).

For the locomotor activity test, an acrylic box (35 cm × 35 cm × 55 cm, black) was set up in another behavioral test room with a dim light. Locomotor activity was recorded via a video camera positioned 150 cm above the central point of the box floor. The imaging data collected were used to measure the traveling distance of each subject, which was calculated using a commercial software (SINGA Real-Time Trace System, version 1.17, Taipei, Taiwan).

The elevated T-maze was set 50 cm above the floor. It was made of wood and had three arms with an equal surface area (50 cm × 10 cm each). The stem of the T-maze was enclosed with 40 cm high walls denoted as the closed arm, which was perpendicular to the two open arms. This apparatus was set up in a behavioral test room separate from those with operant chambers and locomotor activity arena.

### Procedures

Following the adaptation to colony and the cart transportation between colony and behavioral test rooms, all rats were randomly assigned to three groups (*n* = 6 each) to receive acute Ta exposure of  23°C, 28°C, and 35°C for 2 h as the experimental manipulation before the behavioral tests. Following this between-subject design, each rat received a specific Ta exposure throughout behavioral testing. The behavioral tests were conducted in the following order: the elevated T-maze, locomotor activity, FR1 behavior, and DRL 10-s behavior. The Ta conditions in each test room were established 2 h before the commencement of behavioral test. In the test room where the test apparatus was located, each Ta was maintained by means of a reverse-cycle air conditioner. The maintenance of high Ta was run by using an oil-filled radiator heater. Temperature readings taken from two thermometers inside behavioral test room were always within ± 1°C of Ta.

The natural escape and conditioned avoidance to the highness in open-arm area were measured in the elevated T-maze. First, the subject was placed on the far end of the open arm. The escape latency (in seconds) from lingering in the open-arm area to entering the closed area was measured. Second, each rat was placed in the most inside part of the closed area to begin the test trail. The latency of inhibitory avoidance was measured as the time that the rat left the enclosed area. The maximum inhibitory latency was set at 300 s for a single trial. Four trials were conducted to measure the conditioned inhibitory avoidance in the elevated T-maze. A week later, the locomotor activity test was carried out for 30 min by placing the rat into the test arena where the distance (in centimeters) was being measured.

The experiments of operant behavior began 10 days after the locomotor activity test. During this period, the subject adapted to the water restriction regimen. For the first part of the operant behavioral experiment, the rats received 6–10 days of FR1 training where each lever press led a delivery of reinforcer (a water drip in 0.04 ml). The daily session of operant behavioral training and test was 30 min for FR1 or DRL 10-s behavioral task. The criterion to determine the stable performance of FR1 behavior was 120 responses per session that was consecutively observed over 3 days. After meeting this criterion, the subjects underwent a 3-day test of FR1 behavior including a day before and a day after the Ta treatment. No significant difference was observed among the three groups on total responses of FR1 behavior in the pre-test day of Ta treatment (*p* > 0.05; Figure 2A). Subsequently, the subjects remained in the colony for 2 days before entering the second part of the operant behavioral experiment. With a retraining of FR1 response, the rats were then trained to respond on the DRL 10-s schedule of reinforcement, wherein a reinforcer was delivered contingent upon a lever press if at least 10 s had elapsed since the previous press. Premature responses led to a non-reinforcement contingency and a resetting of the interval delay, as indexed by the non-reinforced response. Each lever press, whether reinforced or not, reset the delay timer. The rats acquired the DRL 10-s behavior and reached a baseline in approximately 20 daily sessions of training. The criterion for a stable baseline of DRL 10-s behavior was less than 10% variation in the response rate for three consecutive sessions. Afterward, a 3-day test of DRL 10-s behavior, including a day before and a day after the Ta treatment, was conducted. No between-group difference was observed on the total responses of DRL 10-s on the day before the Ta treatment (*p* > 0.05; Figure 2B).

**Figure 1:**
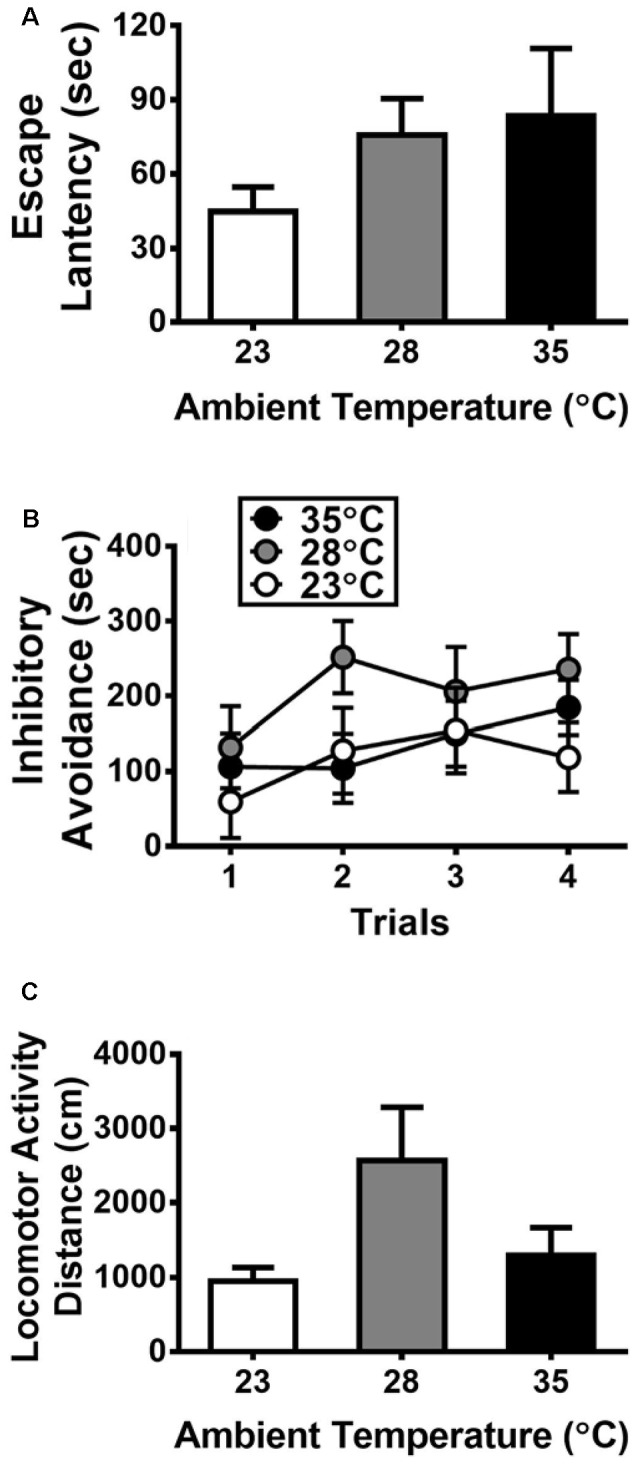
Behavioral responses to exposure of high ambient temperatures (*n* = 6 per group) on the escape latency **(A)** and inhibitory avoidance **(B)** measured in an elevated T-maze and the locomotor activity **(C)** measured in a separate box. All data are displayed as mean ± SEM.

**Figure 2:**
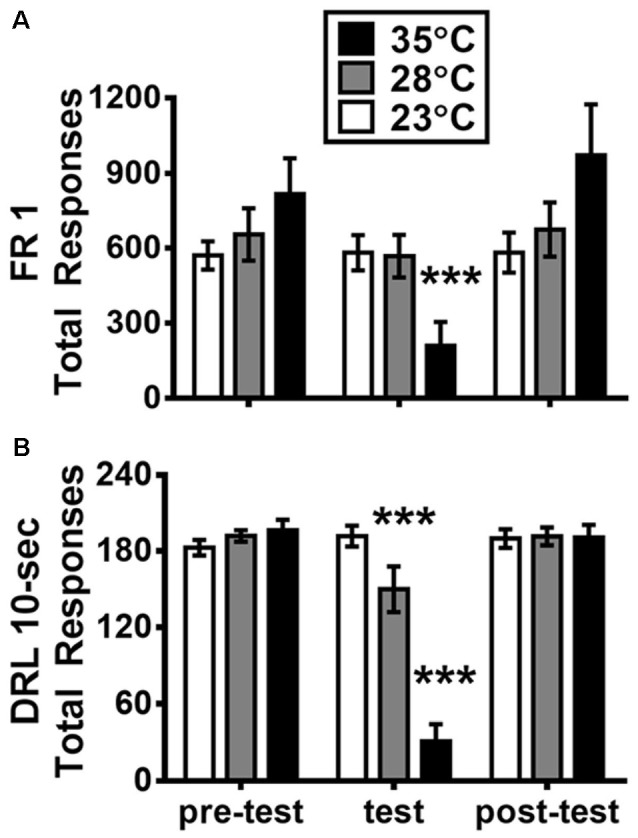
Mean (±SEM) total responses of fixed-ratio 1 (FR1; **A**) and DRL 10-s **(B)** schedule on the days before, during, and after the test of high ambient temperatures. ***p <0.001 (Bonferroni Test) as compared with the pre-test day for each group (*n* = 6).

The core temperature was recorded by using a digital thermometer (HP-100, DRI, Taiwan) with a rectal thermistor probe being inserted to a depth of 30 mm when the rat was lightly restrained. The rectal temperature of each rat was measured immediately before and after the 2 h exposure of Ta, after which FR1 or DRL 10-s behavioral test began. One week after the completion of DRL 10-s behavior test, the subjects of each group underwent Ta treatments for 2 h before being sacrificed by decapitation. Tissues were then collected from the specified brain regions, including mPFC, dSTR, NAcc, AMG, dHIP, and HYPO (Shen et al., 2014). The collected brain tissues were treated with liquid nitrogen and stored in a −80°C freezer until preparation for Western blot analysis.

### Western Blot Analysis

The collected brain tissues were sonicated by lysis buffer (50 mM Tris-HCl, 150 mM NaCl, 2 mM EDTA, 1% Nonidet P-40, pH 8.0) with protease inhibitor Cocktail Set I (Calbiochem) and phosphatase inhibitor cocktail tablets (PhosSTOP). The tissues were then centrifuged at 14,000× *g* for 10 min at 4°C. The lysates were diluted with lysis buffer and the protein concentrations were determined by Bradford assay with a protein assay dye reagent (Bio-Rad). Equal amount of each sample (20 μg) was heated for 10 min at 90°C before loading for Western blot by 10% sodium dodecyl sulfate polyacrylamide gel electrophoresis (SDS-PAGE). The proteins were then transferred onto a polyvinylidene fluoride (PVDF) membrane (Millipore, MA, USA) by wet electroblotting systems. The membrane strips containing the target proteins (according to molecular weight) were cut and immersed in the 0.05% TBS-T buffer (0.05% Tween 20 in Tris-buffered saline) containing 2% bovine serum albumin (BSA, GenDEPOT) for blocking 1 h under room temperature. The strips were then incubated with the primary antibody overnight at 4°C. The primary antibodies included anti-DAT (1:2,000; Abcam, Cambridge, MA, USA), anti-D1R (1:1,000; Santa Cruz Biotechnology, Santa Cruz, CA, USA), anti-D2R (1:1,000; Santa Cruz Biotechnology, Santa Cruz, CA, USA), anti-BDNF (1:1,000; Millipore), and anti-β-actin (1:100,000; Millipore). The membrane was washed in 0.1% Tween 20-TBST for three times (5, 10, and 10 min) and then incubated with either anti-mouse or anti-rabbit IgG-conjugated-horseradish-peroxidase (HRP) secondary antibody (1:5,000) for 1 h under room temperature. Afterward, the membrane was washed again for three times in 0.1% TBST before detection by chemiluminescent reaction with the Immobilon Western chemiluminescent HRP substrate kit (Millipore). The intensity of protein band was quantified by ImageJ (version 1.47, National Institutes of Health, Bethesda, MA, USA) and normalized with the endogenous actin protein as an internal control.

### Statistical Analysis

**Figure 3:**
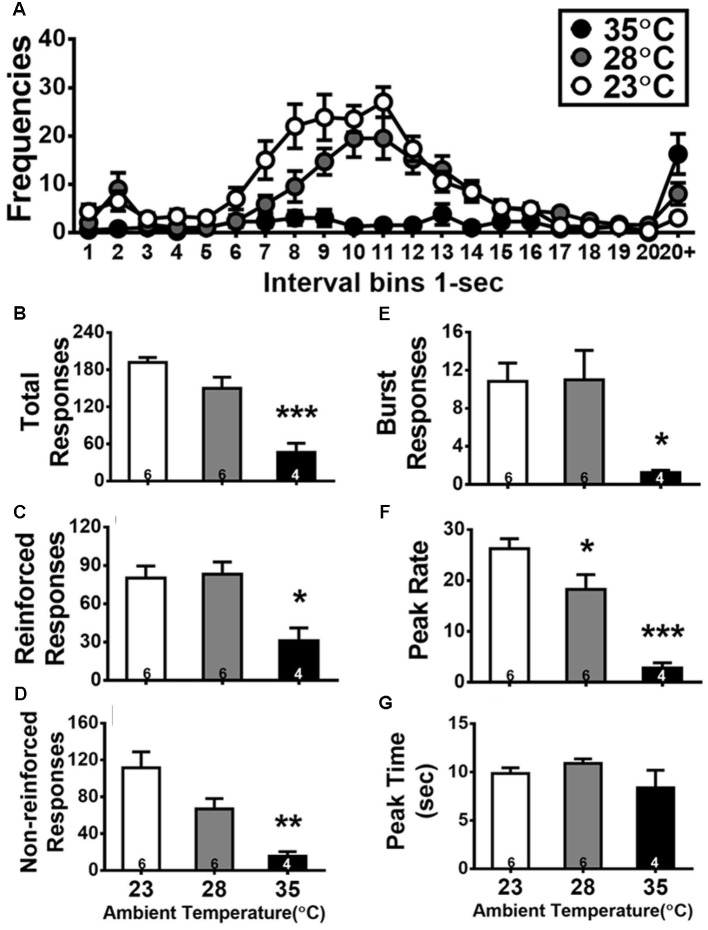
Inter-response time (IRT) distributions of DRL 10-s during the test day of high ambient temperatures **(A)**, and quantitative measurements of total **(B)**, reinforced **(C)**, non-reinforced **(D)**, and burst **(E)** responses as well as peak rate **(F)** and peak time **(G)**. Details of these dependent variables are described in the “Statistical Analysis” section. The number inside the bar (in **B–G**) depicts the sample size of the corresponding group. All data are expressed as mean ± SEM. *p < 0.05, **p < 0.01, and ***p < 0.001 as compared with the control group of 23°C (Bonferroni test).

The number of total responses was recorded for the measurement of FR1 behavior. With regard to the DRL behavior, each lever press was classified in terms of its associated inter-response time (IRT; the time in millisecond elapsed since the prior response). The resulting dataset on IRT was grouped and plotted into a distribution consisting of response frequencies for 21 consecutive 1 s time bins (Figure 3A). For quantitative analyses, six dependent variables were studied: (1) total responses; (2) reinforced responses, lever press with IRT ≥10 s; (3) non-reinforced responses, lever press with IRT <10 s; (4) burst responses, lever response with IRT <2 s; (5) peak rate; and (6) peak time. The burst responses were the summed responses with IRTs that were <2 s (as shown in bins 1 and 2 of the IRT distribution curves in Figure 3A). The peak time and peak rate were calculated from the de-burst IRTs (IRT >2 s), in which a moving average based on four consecutive 1-s bins with a 1-s step size was applied to smoothen the distribution. After identifying the maximum frequencies for a 4-s epoch, the peak time was the average value (in millisecond) of all IRTs that fell within the four bins (i.e., the maximal epoch). The peak time measure indicated at which time point the rats pressed the lever with the highest IRT frequency, i.e., their expected time for obtaining the reinforcer. The peak rate was calculated from the summed responses in the four bins divided by four, indicating how strongly the rats were motivated to press the lever at the expected criterion time. This smoothing procedure has been previously used (e.g., Cheng and Liao, 2007, 2017).

The effects of high Ta exposure on each dependent variable from behavioral measures and biochemical assays were separately subjected to one-way analysis of variance (ANOVA) with the Ta as the between-subject factor. In addition, a two-way mixed ANOVA was used to evaluate the interaction of Ta factor and the test trials or days for certain cases. And, for the effect size calculations partial eta-squared was used ($\eta_{\text{p}}^{2}$). When ANOVA yielded a significant main effect, *post hoc* tests were conducted via the use of Bonferroni correction. Pearson’s correlations were used to examine linear relationships between the measure of operant behavior and each of the protein levels determined by biochemical assay. Statistical significance was set at *α* = 0.05. All analyses were conducted using a computerized statistical program (SPSS version 18.0, SPSS Inc., Chicago, IL, USA). All values are expressed as means ± SEM.

## Results

### Effects of High Ta Exposure on Elevated T-Maze and Locomotor Activity

With regard to the data collected from the elevated T-maze, Figures 1A,B presents the escape latency and inhibitory avoidance, respectively. No significant Ta effect was detected on the escape latency (*p* > 0.05). A two-way ANOVA was used for analyzing the data of inhibitory avoidance, and the results did not yield any significant main effect or interaction (all *p* > 0.05). Figure 1C shows the effects of high Ta on locomotor activity, and there was no significant Ta effect on locomotor activity (*p* > 0.05).

### Effects of High Ta Exposure on the Total Responses of FR1 and DRL 10-s Task

Figure 2 presents the total responses of FR1 and DRL 10-s schedules as measured on the day with high Ta treatment and the days before and after this treatment. For FR1 data shown in Figures 2A, a two-way ANOVA revealed a significant main effect of test day (*F*_(2,30)_ = 22.291, *p* < 0.001; $\eta_{\text{p}}^{2}$ = 0.598) and a significant day-by-group interaction (*F*_(4,30)_ = 15.311, *p* < 0.001; $\eta_{\text{p}}^{2}$ = 0.671). The follow-up simple main effects showed that the group with Ta of 35°C significantly decreased total responses when compared with its own baseline of the pre-test day (*p* < 0.001). For DRL 10-s data shown in Figure 2B, via a two-way ANOVA, the main effects of both test day and group were significant: *F*_(2,30)_ = 78.881, *p* < 0.001 ($\eta_{\text{p}}^{2}$ = 0.840) and *F*_(2,15)_ = 10.541, *p* = 0.0014 ($\eta_{\text{p}}^{2}$ = 0.584), respectively. Additionally, the day-by-group interaction was significant: *F*_(4,30)_ = 45.129, *p* < 0.001; $\eta_{\text{p}}^{2}$ = 0.857. The sample main effects revealed that total responses in both groups of 28°C and 35°C were significantly decreased when compared with their own pre-test day baselines (both *p* < 0.001). As noted, in either FR1 or DRL 10-s, the total responses measured in the post-day test (1 day after the Ta treatment) were not significantly different from those of the pre-test day (*p* > 0.05).

### Quantitative Analyses of the DRL 10-s Data on the Test Day of High Ta Exposure

The effects of high Ta exposure on DRL 10-s task are shown in Figure 3. Figure 3A shows the IRT distribution curves of DRL behavior from the 18 subjects assigned in three groups under different degrees of Ta exposure. A typical bimodal IRT distribution was shown in the control group of 23°C and the group of 28°C. One peak yielded in the bin of 2 s (relevant to burst responses) and another one appeared close to the bin of 10 s. By contrast, the IRT distribution curve was suppressed in the group of 35°C exposure. Quantitative analyses of IRT data of DRL behavior are shown in Figures 3B–G. Two subjects in the group of 35°C emitted less than 5 responses. Considered as the outliers, their data were excluded from quantitative analyses of IRT data for DRL 10-s behavior. Via one-way ANOVAs, the effects of Ta manipulation were significant in five out of six measures: total responses, *F*_(2,13)_ = 23.470, *p* < 0.001 ($\eta_{\text{p}}^{2}$ = 0.783); reinforced responses, *F*_(2,13)_ = 7.543, *p* = 0.0067 ($\eta_{\text{p}}^{2}$ = 0.537); non-reinforced responses, *F*_(2,13)_ = 11.055, *p* = 0.0016 ($\eta_{\text{p}}^{2}$ = 0.630); burst responses, *F*_(2,13)_ = 4.608, *p* = 0.0307 ($\eta_{\text{p}}^{2}$ = 0.415); and peak rate, *F*_(2,13)_ = 22.752, *p* < 0.001 ($\eta_{\text{p}}^{2}$ = 0.778). Following *post hoc* tests, the rats exposed to 35°C exhibited total responses (*p* < 0.001), reinforced responses (*p* < 0.05), non-reinforced responses (*p* < 0.01), burst responses (*p* < 0.05), and peak rate (*p* < 0.001) less than those in the 23°C group. The rats exposed to 28°C performed only a lower peak rate compared with those exposed to 23°C (*p* < 0.05), indicating DRL behavior being slightly but significantly affected by 28°C and profoundly disrupted by 35°C.

As the total responses of DRL decreased for the 28°C and 35°C groups, the other three types of responses (reinforced, non-reinforced, and burst) would be also decreased. Further analysis to normalize each of these three types of responses relative to the total responses was conducted and then tested with one-way ANOVA. The ratio of reinforced responses was increased and that of non-reinforced responses was decreased as Ta went high, but these two trends were not significantly confirmed (both *F*_(2,13)_ = 3.785, *p* = 0.0506). The normalized ratio of burst responses was not significantly changed by Ta, *F*_(2,13)_ = 1.686, *p* > 0.05.

### Rectal Temperature Changes Following High Ta Exposure During Operant Behavioral Tests

As shown in Table 1, a significant Ta-dependent increment of rectal temperature was observed during the FR1 test, *F*_(2,15)_ = 7.779, *p* = 0.005 ($\eta_{\text{p}}^{2}$ = 0.509). From *post hoc* tests, the rats increased rectal temperature when exposed to 35°C (*p* < 0.05). During the DRL 10-s test, although the rectal temperature of the 35°C group was higher than that of the control, ANOVA revealed no significant Ta effect (*p* > 0.05).

**Table 1:** Mean rectal temperature increase (± SEM; °C) from 2 h exposure to 23°C, 28°C, and 35°C (*n* = 6 each group) as measured before the fixed-ratio 1 (FR1) and DRL 10-s behavioral tests.

Behavioral test	23°C	28°C	35°C
FR1	0.85 ± 0.17	0.80 ± 0.28	2.13 ± 0.34*
DRL 10-s	1.37 ± 0.47	1.22 ± 0.16	1.88 ± 0.26

*p < 0.05 as compared with the control group of 23°C (Bonferroni post hoc test).

### Effects of High Ta Exposure on the Protein Levels of D1R, D2R, DAT, and BDNF

**Figure 4:**
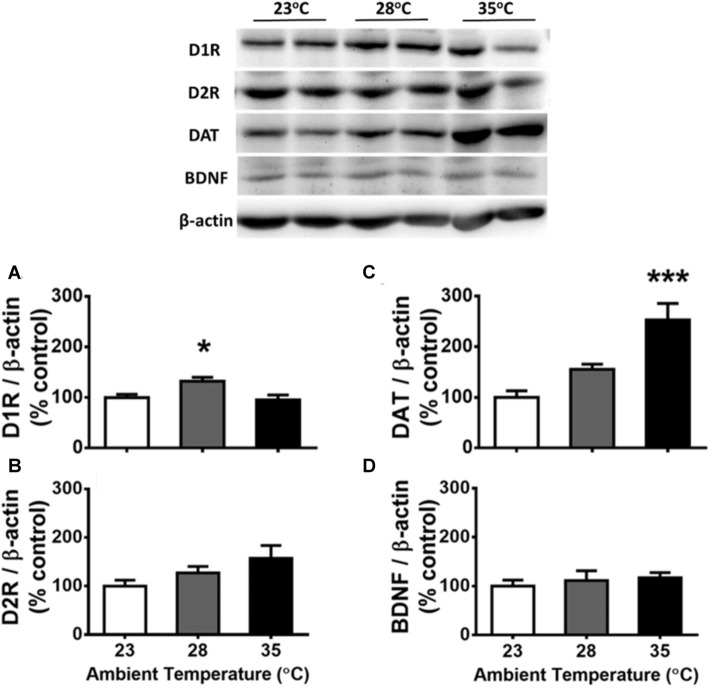
Protein levels of D1 receptors (D1Rs; **A**), D2Rs **(B)**, DA reuptake transporters (DATs; **C**), and brain-derived neurotrophic factor (BDNF; **D**) in the dorsal hippocampus (dHIP) following the exposure of high ambient temperatures (*n* = 6 per group). Representative protein pattern profiles depicting the changes of these four proteins in high ambient temperatures are shown on the *top* panel. All data are displayed as mean ± SEM. *p < 0.05 and ***p < 0.001 as compared with the control group of 23°C (Bonferroni test).

The results corresponding to the dHIP are presented in Figure 4. Using one-way ANOVA, significant Ta effects were detected on the protein levels of D1R (Figure 4A) and DAT (Figure 4C) in the dHIP, *F*_(2,15)_ = 6.292, *p* = 0.0104 ($\eta_{\text{p}}^{2}$ = 0.456) and *F*_(2,15)_ = 2.423, *p* < 0.001 ($\eta_{\text{p}}^{2}$ = 0.638), respectively. Compared with the control of 23°C from *post hoc* tests, the 28°C and 35°C treatments increased in D1R (*p* < 0.05) and DAT (*p* < 0.001), respectively. No Ta effect was observed on D2R (Figure 4B) or BDNF (Figure 4D) in the dHIP (*p* > 0.05). There was a significant correlation between the DAT level and total responses of FR1 (*r*_(16)_ = −0.510, *p* = 0.031) and so was for that of DRL 10-s (*r*_(16)_ = −0.762, *p* < 0.001), indicating lower operant responding is associated with higher level of DAT expressed in the dHIP after high Ta exposure. The relationship between the total response of either operant task and the dHIP D1 receptor level was not significantly confirmed (both *p* > 0.05). Furthermore, this type of negative correlation was significantly appeared for the dHIP DAT, but not D1 receptor, level in associated with quantitative measures of IRT data for DRL 10-s behavior including the reinforced responses (*r*_(14)_ = −0.674, *p* = 0.004), the non-reinforced responses (*r*_(14)_ = −0.733, *p* = 0.001), the burst responses (*r*_(14)_ = −0.536, *p* = 0.032), and the peak rate (*r*_(14)_ = −0.803, *p* < 0.001). That relationship corresponding to the peak time was not significant (*r*_(14)_ = −0.493, *p* = 0.052).

In contrast to the results of the dHIP, no significant Ta effect was confirmed for each protein level expressed in the other five brain regions (all *p* > 0.05; data not presented).

## Discussion

Behavioral changes following high Ta exposure have been mostly revealed by the use of tasks related to the reflexive, but not conditioned, type of behavioral response. Here, we show that acute exposure to high Ta (28°C and 35°C as compared to the control of 23°C) produced different profile changes on two types of operant behavior respectively maintained on the FR1 and DRL 10-s schedule of reinforcement. While the total responses of FR1 behavior were decreased only under 35°C when compared with the control group of 23°C, those of DRL 10-s behavior were significantly reduced in both groups of 28°C and 35°C. Distinct patterns of IRT distribution of DRL behavior appeared among the three groups; between-group differences of behavioral changes produced by high Ta exposure were confirmed by quantitative analyses of IRT data. In addition, via Western blot assays, the Ta-related increments were found in the protein levels of D1 receptors and DAT expressed in the dHIP.

### Differential Effects of Two Levels of High Ta on FR1 and DRL 10-s Behaviors

In this study, operant responses to FR1 and DRL 10-s schedules were significantly affected by the acute exposure to high Ta being manipulated in 28°C and 35°C. The changes were more profound in operant responses to DRL schedule than that in the FR one. The operant responses of DRL 10-s behavior significantly decreased in both the 28°C and 35°C groups when compared with their corresponding baselines given in the pre-test day (Figure 2). For the FR1 test, a significant decrease in total responses was detected only in the 35°C group. In other words, acute exposure to 28°C affected operant response to DRL 10-s schedule, whereas the performance of FR1 schedule was preserved. These results indicate that the performance operant behavior can be distinctively affected by acute exposure of different degrees of high Ta and also in a task-dependent fashion. The results presented herein contribute to a further understanding about how the performance of operant behavior can be affected by acute exposure to high Ta. While the literature on this topic is scarce, exposure to 38°C has been reported to decrease the response rate measured in both FR10 and DRL 18-s components of a multiple DRL-FR schedule-controlled behavior (Thomas et al., 1991). In the other study by Barofsky (1969), during the exposure to 35°C, response and reinforcement rates of DRL 15-s behavior profoundly decreased to a minimum level within 40–50 min of the 90 min test session. Despite that a significant adverse effect on both FR and DRL tasks was consistently observed in high Ta exposure of 35°C or 38°C, some methodological discrepancies (e.g., experimental design) warrant consideration for comparing the results between studies. First, descriptive, but not inferential, statistics was presented in previous studies (Barofsky, 1969; Thomas et al., 1991). The use of rather small sample size (*n* = 4) inevitably forced the experiments run in a within-subject design for testing both FR and DRL behaviors over multiple conditions of thermal stress, even from cold to warm Ta (Thomas et al., 1991). Second, only one degree of high Ta was tested in comparison with the control Ta (e.g., 38°C vs. 24°C or 35°C vs. 25°C) in previous studies. In contrast, data in the current study were collected from a between-subject design for the exposure to 23°C, 28°C, and 35°C across four behavioral tests including the separate tasks of FR and DRL operant behavior. The current data are deemed to be informative for clarifying the previously reported results that were influenced by certain confounding factors. Importantly, in the present study, the performance of DRL behavior was significantly affected by acute exposure to a low level of high Ta given in 28°C, whereas that of FR behavior was left intact. These behavioral changes may be associated to characteristic differences between DRL and FR schedule-controlled behaviors as mentioned in the Introduction. That is, as compared with the FR task, the DRL task known with a higher degree of task difficulty that requires for behavioral inhibition and timing process. More sessions are normally needed to train operant response in DRL schedule than that in the FR schedule before reaching an acceptable baseline. It is then possible that a cognitively demanding DRL behavior is more vulnerable to be affected by high Ta exposure than a simply lever-pressing FR behavior.

It should also be noted that the aforementioned effects of acute exposure to high Ta on operant behaviors were short-term, because the behavioral performance that was measured in the post-test day returned to the level of the pre-test day for either FR or DRL task. The present findings that the adverse effects of DRL 10-s behavior induced by 35°C treatment reversed to its baseline level is consistent with the report by Barofsky (1969). Interestingly, differential effects of two levels of high Ta exposure (i.e., 30°C and 35°C) have been recently observed in a food-intake test for 1 h but not daily (Suwanapaporn et al., 2017).

### Negative Effects of High Ta on Locomotor Activity and Elevated T-Maze Test

The above-mentioned changes of operant behaviors cannot be ascribed to the alteration of gross motor function or anxiety-like response following acute exposure to high Ta. Statistical analyses revealed that the locomotor activity measured in the group of 28°C or 35°C was not significantly different from that of the control group of 23°C, despite the presence of an incremental trend. The null result of present high Ta exposure on locomotor activity is similar to that of the vehicle control subjects exposed to 27°C (vs. 23°C) or 30°C (vs. 20°C) as reported in previous studies, which tested the effects of high Ta on thermoregulatory and hyper-locomotion responses to psychoactive drugs (Wright et al., 2012; Miller et al., 2013). Intriguingly, a decrease in locomotor activity at 30°C (vs. 19°C) has been reported in vehicle-treated rats, which were compared with 3,4-methylenedioxymethamphetamine treated subjects (Hargreaves et al., 2007). No severe depression of locomotor activity (e.g., akinesia) was observed in the rats of 28°C and 35°C groups in the present study. Thus, the profound decrease in total numbers of responses measured in either FR1 or DRL 10-s were not attributed to locomotor activity disrupted or suppressed by high Ta exposure.

The exposure to high Ta is a kind of stressor, and the stress is known as a precipitating factor for anxiety-related behavior and disorders. Therefore, the appearance of anxiety or panic attacks may increase as the temperature also increases. However, contrary to this notion, the present high Ta exposure did not affect the escape latency and inhibitory avoidance as measured in the anxiety-like responses in the elevated T-maze. By using this task, the decrement of escape latency and the increment of inhibitory avoidance have been shown to model panic and general anxiety disorders, respectively, in the rat (Zangrossi and Graeff, 2014). During the present test of escape latency (Figure 1A), the subjects in the experimental groups with high Ta exposure went into the closed area with a latency a bit longer than the control subjects, but there was no between-group difference. Also, a null result of high Ta exposure was obtained for the test of inhibitory avoidance. Thus, these data reflected no detectable anxiety-like response in this task for the rats of 28°C and 35°C groups compared with the controls in this study. The performance of the control subjects on elevated T-maze in the present study is akin to that of the normal subjects being reported by this laboratory and the others (Silveira et al., 2001; Chang and Liao, 2005). Nevertheless, it is noted that various types of animal models with distinct behavioral constructs and brain mechanism have been used in the field of anxiety research with certain debated issues being concerned (Ennaceur, 2014; Lezak et al., 2017). Thus, caution should be taken in reading this part of the results regarding the negative effects of high Ta exposure on the anxiety test using the elevated T-maze. More research is required before any conclusion can be made to elucidate this issue.

### Neural Basis of Operant Behavior Altered by High Ta Exposure

The phenomenon where body temperature increases when an organism is confronted with a stressor is known as stress-induced hyperthermia. This hyperthermia phenomenon has been observed even as mildly induced by an injection (Olivier et al., 2003) or psychological threat (Nakamura, 2015) in the rodent. Given high Ta exposure as a stressor, the increase of rectal temperature after the high Ta exposure was significant in the subjects of the 35°C group of the present study. However, this effect was not the case for the subjects of the 28°C group. In fact, the change in rectal temperature of the 28°C group was smaller than that of the control group (despite no significant difference). This result, nonetheless, supports the argument that the effects of different levels of high Ta exposure can be dissociable on behavioral and physiological aspects (Leon et al., 2010; Suwanapaporn et al., 2017). The reason why the significant Ta-related increase of rectal temperature appeared in FR1, but not DRL 10-s, test is unclear. The adaptation to high Ta exposure given in multiple trials (i.e., the third time in FR1 test and the fourth time in DRL 10-s test) may be related to this issue. More research is needed to confirm this hypothesis.

Conversely to the adaptation view, a possibility remains that the observation of two operant behaviors changed by high Ta, but no significant Ta effect in the tests of elevated T-maze and locomotor activity, may be due to the testing order. Namely, multiple sessions of high Ta exposure (as a stressor) over time could have carryover effects into the subsequent tests. Exposure to stressors (e.g., disrupted sleep, predator odor stress, etc.) can sensitize the animal’s responses (e.g., elevated HPA axis output, increased anxiety, deficits in cognition, etc.) to future stressors. We acknowledge several limitations in this study for tackling this issue. But just with the rectal temperature measured for two operant behavioral tests, if this issue is the case, then the rise of rectal temperature would be expected to be consistently higher in DRL 10-s test than that in FR1 test. A two-way ANOVA (3-group-by-2-test) revealed a significant group effect, *F*_(2,15)_ = 6.113, *p* = 0.011, neither the test main effect nor the interaction was significant (*p* > 0.05). The hypothetical increase of body temperature along with the repeated exposures to high Ta may be depedentent on experimental condition. Muskherjee and Poddar (2001) reported that rectal temperature was significantly increased by a daily 2 h exposure of 40°C for 30 consecutive days in their normal control rats. In the present study, the exposure to high Ta were manipulated for five times in total, which were separated in 1 week or longer. Thus, given the carryover effect might be potentially existed, the effectiveness could be subtle.

In addition to the central circuitries for body temperature regulation (Nakamura, 2011), the midbrain DA systems have been indicated to be critically important for autonomic and behavioral thermoregulation (Brown et al., 1982; Lee et al., 1985). Nevertheless, how Ta affects or modifies the DA-involved thermoregulatory system is largely unknown to date. The enhancement of DA release has been shown in the rodent under various kinds of stressors (Roth et al., 1988; Feenstra, 2000). In absence of neurochemical evidence regarding the synaptic levels of DA directly examined in the rat under high Ta exposure, a growing body of research suggests that increased central DA activity including the neurotransmitter release is essential for physical activity (e.g., exercise) linked with hyperthermia (Zheng and Hasegawa, 2016). Therefore, exposure to high Ta can possibly enhance the release of DA in terminal areas of brain DA systems. Such DA enhancement may cause changes in postsynaptic receptors and presynaptic reuptake transporter in confronting the impact from high Ta challenge. In this study, via a biochemical assay, the expressions of D1 and D2 receptors as well as DAT were shown in distinct patterns following high Ta exposure. In terms of the Ta-dependent effect, we found that the protein levels of D1 receptors and DAT were significantly increased in the dHIP in the 28°C and the 35°C groups, respectively. Despite the appearance of Ta-related increment in D2 receptors of the dHIP, it was not significantly confirmed by statistical test. In contrast to the results of dHIP, those of D1 and D2 receptors and DAT expressed in the other five regions were not significantly detected in high Ta treatment. That the acute exposure to high/warm Ta had a significant adverse effect on FR1 and DRL 10-s behaviors may be involved with stress-induced hyperthermia by increasing the protein levels of DAT expressed in the dHIP following the synaptic release of DA increased by temperature stress. This notion is supported by the negative correlations between the DAT level and all behavioral measures except the peak time of DRL test. In this region, the observation of Ta-related increment of DAT implicates that the presynaptic reuptake mechanism is critical for the neural adaptation following the impact of high level of warm Ta (e.g., 35°C). Conversely, the postsynaptic D1 receptors may be more sensitive to alterations during low levels of warm Ta (e.g., 28°C). Regarding the role of the hippocampus in the DRL behavior, previous studies showed impaired performance of a DRL 12-s task in hippocampectomized rats (Rawlins et al., 1983) and deficits in acquiring a DRL 18-s behavior after cytotoxic hippocampal lesion (Bannerman et al., 1999). Moreover, with neurophysiological recording, the vast majority of hippocampal cells displayed event-related profiles in association to DRL 15-s behavior (Young and McNaughton, 2000). Together, the altered expressions of D1 receptor and DAT in the dHIP may be associated with the adverse effect on DRL 10-s behavior produced by high Ta exposure. Further investigation is needed to elucidate the cause-effect mechanisms underlying this proposed relationship. One more issue worth noting is the present neurochemical data were obtained in separate to behavioral experiments (i.e., after at the end of behavioral testing). It could potentially reflect a summed effect of all five-time high Ta exposure. A factorial design of high Ta exposure on both operant behaviors with brain samples collected immediately after behavioral testing may be applied to tackle this issue.

With regard to BDNF, no detectable Ta-related effect was revealed in any of the six areas tested in this study. The absence of BDNF change could be due to the degree of high Ta exposure in this study is lower than that typically applied in other studies of heat shock or heat-related illness (e.g., >40°C; Sharma and Johanson, 2007). It is also possible that BDNF is associated to the chronic, but not acute, exposure to high Ta. The levels of BDNF have also been shown to be altered by the long-term exposure to high Ta in adult rats (Matsuzaki et al., 2017) and in chicks during their postnatal stage (Katz and Meiri, 2006). Thus, the acute exposure, rather than chronic/long-term manipulation, of high Ta applied in this study may be attributed to this null-effect outcome.

## Conclusion

Our data indicate that the performance of operant behavior can be distinctly affected by exposure to different degrees of high Ta in a task-dependent fashion. The operant behavior maintained on DRL task is more sensitive to be altered than that of the FR task by high Ta exposure. These behavioral changes are not the result of motor suppression or anxiety-like response induced by high Ta exposure. The change of DAT in the dHIP is associated to these changes of operant behavior. The current results complement those from studies in concerned with the physiological and behavioral thermoregulation mechanisms and provide a better understanding of the effects of high Ta exposure on operant conditioned behavior.

## Data Availability

All datasets generated for this study are included in the manuscript.

## Author Contributions

S-FC, C-YChu and R-ML conceived and designed the experiments. S-FC, C-YChuang, Y-HY, C-YChu, C-YY, Y-CS and Y-HH performed the behavioral experiments. S-FC, C-YChuang and C-CC ran biochemical assay. S-FC, C-CC and R-ML analyzed the data and contributed to writing the manuscript.

## Conflict of Interest Statement

The authors declare that the research was conducted in the absence of any commercial or financial relationships that could be construed as a potential conflict of interest.
